# The Contribution of Bayesian Methods in Solving the Paradoxes of Classical Statistical Tests in Biomedical Research

**DOI:** 10.3390/jcm15062262

**Published:** 2026-03-16

**Authors:** Nicolas Meyer

**Affiliations:** 1 GMRC, Groupe Méthodes en Recherche Clinique, Service de Santé Publique, Hôpitaux Universitaires de Strasbourg, 67000 Strasbourg, France; nicolas.meyer@chru-strasbourg.fr; 2 iCUBE, UMR 7357, Laboratoire des Sciences de L’ingénieur, de L’informatique et de L’imagerie, 67412 Illkirch, France

**Keywords:** null hypothesis test, hypothesis test, Neyman–Pearson, significance test, Fisher, *p*-value, Bayesian statistics

## Abstract

Almost all publications in biomedical literature have employed statistical tests, with *p*-values being considered of particular importance in the assessment of the presence of a link between two variables. However, these tests and *p*-values have been the subject of considerable criticism. It may appear paradoxical that tools utilised by the scientific community for nearly a century could possess all the flaws attributed to them. This paradox can partially be explained by the counterintuitive nature of *p*-values and the fact that the test that generates them is the result of a combination of two tests that were developed to answer statistical questions of a very different nature. The respective characteristics of these two tests are essentially unknown to the majority of users of *p*-values. The aforementioned paradox can be partially explained by the paucity of publications that seek to elucidate these concepts for users of *p*-values, the majority of whom are not statisticians. The recently introduced Bayesian methods have properties that enable us to understand the limitations of traditional methods. In Bayesian methods, the use of a specific interpretation of probability allows for better exploitation of clinical research data. The aim of this article is to highlight the limits of non-Bayesian methods and explain the principles and functioning of Bayesian methods to a non-statistical audience.

## 1. Introduction

Statistics are everywhere in medicine, including in therapeutic trials, in epidemiology, and in the evaluation of diagnostic tests. Virtually every publication contains a statement such as the following: “In a comparison with placebo in the treatment of disease X, we observed a significant difference in success rate in favour of treatment A of 6% [1.9–10.6], *p* = 0.001.” Such results are obtained using what is known as the null hypothesis test (NHT), a statistical tool that provides the famous *p*-value. Although it has become indispensable, the NHT has nevertheless been severely criticised over the last 20 years [1,2,3,4,5,6,7,8,9,10,11,12,13,14], and it is therefore legitimate to wonder why a method used so often and by so many authors can be so disparaged. It is true that the NHT and *p*-values are not easy to understand and are very often misinterpreted and misused [15,16,17,18,19,20,21]. Many investigators have encountered issues such as alpha risk correction, multiple tests, and sequential analyses, which may require different modifications in different contexts. The justifications for these modifications are often obscure and sometimes contradictory from one statistician to another. Additionally, the *p*-value can change significantly depending on the type of test, the addition or removal of subjects from the analysis, or missing data [22]. Under the “publish or perish pressure”, most authors feel compelled to provide significant *p*-values [4,5]. The perceived need of investigators to use NHT and *p*-values to validate their work thus leads to an almost mechanical and ritualised use [23,24] of these methods, which more or less hides their lack of understanding of how they work. Therefore, what should be done? Should we continue to use them? Modify them? Or move on to other methods?

The confusion has recently worsened with the emergence of Bayesian methods, particularly in the analysis of therapeutic trials [25]. Although these methods are now accepted by both journal editors and regulatory bodies, very few clinicians are aware of what they involve. There are already several publications on the subject [26,27,28,29,30,31,32,33,34,35,36], but these articles are often too complex for their intended audience, and, above all, they do not clearly explain why Bayesian methods would be more useful than conventional tests, nor why these methods should replace conventional methods that seem to work so well, despite the criticism levelled against them. These articles generally focus on the more technical aspects of Bayesian methods and do not clearly explain the underlying concepts, such as the different interpretations of probability or why the NHT is not in fact the ideal statistical tool in biology or medicine. While statistic users may eventually be convinced to use Bayesian methods rather than non-Bayesian methods, too superficial an understanding of the underlying principles could lead to one ritual being substituted for another, replacing one misuse with another. For researchers to truly embrace Bayesian methods, it seems important that they have a detailed understanding of the underlying details.

The aim of the present article is, therefore, first, to show that the NHT is a procedure that incorrectly combines two tests designed to deal with very different problems [17,18,19,20,21]. This will enable the reader to understand the origin of the multiple paradoxes of the NHT and its misuse, and why it is so difficult to avoid falling into the trap of this misuse. Then, the different interpretations of the concept of probability will be presented, which will provide a better understanding of the principles of both non-Bayesian (i.e., classical) and Bayesian methods. Finally, we will explain why Bayesian methods allow for better interpretation of data from biomedical research.

## 2. The Fundamental Principles of Classical Statistics

Biology and medicine are sciences that aim to establish knowledge about the state of nature, for example, by identifying a pathophysiological mechanism or demonstrating the superiority of one treatment over another in general terms, i.e., for any subject in a population. This assumes that there is a true and unique although unknown value in nature of what we are trying to measure (a true odds ratio, or a true mean difference). However, since the population is, by definition, infinite in size, it cannot be studied in its entirety, and the true value of what is measured cannot be obtained directly. We must, therefore, use a sample extracted from this population of interest to establish knowledge about the value of this measurement, i.e., estimate its true value. However, since this sample is obtained by a random mechanism, the value observed in the sample will inevitably be more or less different from the true value for the population. There is, therefore, some uncertainty about the exact value of the measurement in the population. This is why we must use the laws of statistics, which allow us to infer the value of the measurement sought in the population from the value observed in the sample.

To establish the possible superiority of treatment A over treatment B, all current publications rely on the NHT. This is a two-step procedure. The first step is to formulate the null hypothesis that the two treatments have the same efficacy. Statistical principles can then be used to predict the entire range of differences that may be observed between the two groups if this assumption is true. Next, the difference observed in the sample is compared with these expected difference values. If the observed result is highly unlikely, i.e., if its probability *p* of obtaining a difference as large as the observed one is below a pre-set threshold (most commonly called alpha and set equal to 0.05), the null hypothesis is considered false, and the test is said to be significant. This leads to the conclusion that there is a difference in efficacy between the two treatments. Conversely, if *p* > alpha, the hypothesis of no difference is not rejected, which implies that this non-rejection is only temporary. In all cases where H0 is rejected, it is implied that the difference applies to the target population. For example, Dequin et al. [37] evaluated the benefits of hydrocortisone in severe community-acquired pneumonia (CAP). In the study, patients hospitalised in intensive care were randomised to either hydrocortisone or a placebo, in addition to standard treatment. The death rate at 28 days was observed to be lower in the hydrocortisone group, with an absolute difference of −5.6% [−9.6 to −1.7] and a *p*-value of 0.006, which was lower than the chosen risk of 5%. The authors concluded that hydrocortisone is effective, suggesting that this is a general result applicable to all patients with severe CAP hospitalised in intensive care, i.e., in the population of severe CAP.

As strange as it may seem, the NHT procedure described above has never been theorised as such by any statisticians [4,6,20,21]. It is a kind of self-constituted hybrid based on elements from two other tests that have been formally established and studied: the Neyman–Pearson hypothesis test (HT) [38,39] and Fisher’s significance test (ST) [40]. The problem is that these two methods were developed for very different situations, which gave rise to their specific characteristics and a rather different interpretation of their results. It is true that these differences are very subtle, which has facilitated their hybridisation into a procedure that resembles both methods while having no solid mathematical basis and, above all, no scientific validity. The fact that everyone uses this procedure, however, poses a major challenge to anyone who wants to dismantle its mechanism, resulting in a vicious cycle. Since everyone uses this method, it must be the right one; if it is the right method, it must be taught; since it is taught everywhere, it must be the right method to use. Consequently, everyone uses it [13].

It is, therefore, necessary to analyse the functioning of the two tests mentioned to understand what distinguishes them and why their combination constitutes an unsuitable tool for its intended use.

### 2.1. The Neyman–Pearson Hypothesis Test

The history of science is useful for understanding scientific theories. Neyman and Pearson explicitly state in their 1933 article [38,39] that they consider Bayesian methods, already known at the time, to be the only ones that can be used to address scientific problems. However, they were concerned about certain epistemological consequences of these methods (see below) [39]. Furthermore, Neyman and Pearson would occasionally work for industrialists, who have very specific statistical problems relating mainly to quality control (QC) on production batches. It was for this purpose that Neyman and Pearson explicitly (albeit very discreetly, in a brief footnote [39]) developed their theory of statistical testing. They then devised a method for identifying compliant and defective batches using a statistical test, but without resorting to Bayesian principles. To do this, they relied on the typically frequentist notion of test repeatability: each of the thousands of batches manufactured would be tested and classified as compliant or defective on the basis of a sample. The same characteristic is tested repeatedly on each batch, which are objects of the same type under stable conditions. The performance of the classification is assured over the long term rather than on each sample.

Indeed, the main problem for a manufacturer is to market as many of its production batches as possible, which are inherently repeatable, similar, and numerous. The manufacturer must be able to determine from a sample taken from a given batch whether the batch in question complies with the production standard (H0) and whether it can be marketed or whether it must be withdrawn due to non-compliance of the batch (H1). This problem requires a decision between two possible actions: market or discard. It is not a question of acquiring general knowledge about the production line’s ability to supply compliant batches, but rather of treating each individual batch tested as either compliant or non-compliant and acting on each batch according to the classification made by the test. It is for this very specific context that Neyman and Pearson developed what would later be called hypothesis testing (HT), which explains why they described their test as a “behaviour-inducing decision-making procedure” [39].

For example, let us assume that a company manufactures 250 mg amoxicillin (Am) tablets. A quality control (QC) test is carried out to ensure that the tablets contain 250 mg of Am. A margin of 5 mg above or below this amount is tolerated, which defines a compliant batch as one in which the average Am content is between 245 and 255 mg. The company produces batches of 10,000 tablets every hour. Each batch is either compliant (H0) or non-compliant (H1) with this standard. The quality engineer must check the mean amoxicillin content of the tablets in each batch. As it is not possible to test all 10,000 tablets in each batch, he takes a sample of 20 tablets and tests them. He chooses a test (Student’s *t*-test, for example) to compare the average amount of Am in the sample tablets with the value of 250. He calculates the average Am in the sample and performs the required statistical test. If the statistical value is beyond the tolerance margin (to simplify, if the *t*-test suggests that the mean content is larger than 255 or smaller 245 mg), he will classify the batch as H1 and suggest that it should not be marketed. If the value of *t* is within the acceptable limits, he will classify the batch as H0, which will allow it to be marketed. Each batch must be tested, and knowledge about a given batch does not provide any information about another batch, which must be tested on its own. It is obviously possible to make a mistake when classifying a sample (by deciding that a valid sample should be withdrawn from sale or vice versa), and one of the strengths of the Neyman and Pearson method is that it allows the classification error rate to be controlled. This is the origin of the famous alpha and beta risks. However, as well-known as they are, these risks are systematically misunderstood and confused with the probability that the null hypothesis is false.

Based on the usual risks levels seen in the literature, 5% (alpha%) of the compliant batches will be incorrectly rejected as non-compliant, and 80% (1-beta) of non-compliant batches will be correctly identified by this test. All of this seems perfectly consistent with everything everyone has learned about testing. However, the most subtle point of the Neyman and Pearson method is undoubtedly its paradoxical nature. When applied in a scientific context, it remains valid and perfectly respects the constraints imposed on it; however, it does not provide the expected answer, namely whether the tested hypothesis is actually credible or not. Whatever the true frequency of compliant batches, the proportion of correctly classified compliant batches will be high (at least 80%), but it provides no information about the true frequency of compliant batches. While the manufacturer’s problem is to classify each batch with as little error as possible, the scientific formulation of the quality control problem would be to try to establish the frequency, i.e., the probability of the two types of batches. The aim of a researcher evaluating a treatment is not to classify a particular therapeutic trial on a given sample of patients as significant or not, but to draw a valid general conclusion on the treatment effect for the whole population of patients with the condition being treated. However, unexpectedly, the Neyman and Pearson procedure does not provide this information. It is undoubtedly this phenomenon, which is difficult for the user to perceive, that is at the root of the greatest misunderstanding in the use of statistical tests in medicine and biology. What, then, is the problem?

The problem is as follows: Let us assume that the production line operates correctly in 98% of cases. Then (Table 1), among 980 compliant batches, 49 (5%) will be classified as non-compliant by a significant test. Among the 20 non-compliant batches, 16 (80%) will be correctly identified by a significant test. Most of these significant tests (49 out of 65) will therefore actually be attributable to false alarms (false positives) on compliant batches. Now suppose that the machine only works correctly half the time. Then, the majority of significant tests will mainly come from non-compliant batches since 400 of the 425 with significant results come from the 500 non-compliant batches. So, again, where is the problem? The problem is that in these two very different situations, in terms of the frequency of compliant and defective batches, the alpha and beta risks are fully respected. However, when testing a batch, whether the results are significant or not, we cannot deduce the frequency of compliant versus defective batches. Therefore, it is not possible to know whether the production line mainly produces compliant batches (first situation) or defective batches (second situation). Obtaining a significant test on a sample from a batch does not allow us to determine, even probabilistically, whether the batch is actually compliant or not. The only information that this test provides—and this is known even before it is applied—is that regardless of the frequency of compliant batches, 80% of defective batches will be correctly classified, and 5% of compliant batches will be incorrectly classified. Using the test in reverse is invalid: a batch classified as compliant provides very little indication of the probability that the batch is actually compliant. Whatever this probability may be, and similarly to the sensitivity and specificity of a diagnostic test, the alpha and beta risks are intrinsic properties of the test. They are not properties of either the sample or the hypotheses, and these risk levels do not represent the probability that each hypothesis is true or false.

Transposed into the context of clinical trials, this means that obtaining a significant test does not indicate the probability that the hypothesis of equal treatment effects is true. Therefore, in a therapeutic trial comparing two treatments A and B, a significant test will not allow us to conclude in general terms that A is more effective than B, as the result is only valid for the sample tested and cannot be generalised to the population of patients to be treated.

To compare two treatment groups, for example, the quality control Neyman–Pearson procedure would formulate two hypotheses: a null hypothesis (H0) and an alternative hypothesis (H1). The first specifies an absence of differences between the compared groups, and the second, which is essential to the procedure, defines a difference of at least delta units against which the test is performed. The procedure also requires choosing an alpha risk and a beta risk, on which the power 1–beta depends. Depending on the type of variable to be compared between groups, a test statistic (chi^2^, Student’s *t*, or other) is identified, which will support the decision. Based on alpha, beta, and delta, a threshold value for the test statistic is determined, which, in turn, defines a rejection zone for H0 and an acceptance zone for H0 for the test. For example, for an alpha risk of 0.05 and a sample size of 30 subjects per group, the threshold value for Student’s *t*-test is approximately equal to 2. When the test statistic (Student’s *t*, chi^2^, etc.) is greater than the threshold value, H0 is rejected and it is concluded that hypothesis H1 should be retained; otherwise, H0 is retained. The significance of this conclusion is investigated in more detail later. Applied to the cortisone example, the chi^2^ test would have a value of 4.6, which is greater than the threshold of 3.84, leading to the conclusion that there is a difference in efficacy between the treatments. However, as shown above, this significant result could just as easily have been generated by a sample from a population in which cortisone is effective as by a sample from a population of patients in which cortisone is ineffective, thus generating a false positive. Unfortunately, it is not possible to distinguish between these two situations on the basis of the significant result alone.

The method of Neyman and Pearson is called a hypothesis test, but it is actually more of a procedure for classifying batches according to two hypotheses, as its designers intended. Neyman and Pearson were quite explicit on this point: “*But we may look at the purpose of tests from another view-point. Without hoping to know whether each separate hypothesis is true or false, we may search for rules to govern our behaviour with regard to them, in following which we insure that, in the long run of experience, we shall not be too often wrong. Here, for example, would be such a “rule of behaviour”: to decide whether a hypothesis, H, of a given type be rejected or not, calculate a specified character, x, of the observed facts; if x > x_0_ reject H, if x ≤ x_0_ accept H. Such a rule tells us nothing as to whether in a particular case H is true when x ≤ x_0_ or false when x > x_0_. But it may often be proved that if we behave according to such a rule, then in the long run we shall reject H when it is true not more, say, than once in a hundred times, and in addition we may have evidence that we shall reject H sufficiently often when it is false*” [39]. They were not looking for a method to validate a scientific hypothesis (on the true rate of compliant batches on the production line), but rather to determine what to do with each batch produced by the line, with an acceptable error rate in decisions regardless of the true rate of compliant batches. Therefore, it is more accurate to refer to this as batch testing based on two hypotheses rather than hypothesis testing. The hypotheses as such are not really tested, and the results of the test do not change the knowledge about them. The test does not provide any knowledge about the veracity of these two hypotheses because the Neyman–Pearson test is constructed in such a way that it does not require any knowledge on the veracity of the hypotheses to run. The test operates as a diagnostic test that does not require knowledge of the prevalence of the disease to classify a subject with a positive test result as ill. When positive, we *act* as if the subject is indeed carrying the disease. However, if we want to estimate the probability that the subject is indeed ill by calculating the positive predictive value, we need to know the disease prevalence. Just as the diagnostic test does not provide the prevalence of the disease in the population based solely on the observation of a positive test result, the Neyman–Pearson HT, regardless of its conclusion, does not provide the probability that H0 and H1 are true [19].

Since the aim is to make a practical decision based on the test result (such as whether or not to market the tested batch), it is also understandable why, in the Neyman and Pearson procedure, one may be led to truly accept H0. When a sample is classified as coming from an H0 batch, this conclusion must be adhered to for the resulting action, i.e., marketing the corresponding batch that is deemed compliant. This contrasts sharply with the attitude of ostracism and suspicion towards non-significant H0s that prevails throughout the biomedical literature.

This is an extremely powerful cognitive bias that causes us to mistake the probability of a significant test result when the batch is non-compliant (i.e., the power of the test) for the probability of a non-compliant batch when the test result is significant (i.e., the probability that H1 is true). However, the probability of having hypertension when you have a pheochromocytoma is very different from the probability of having a pheochromocytoma when you have hypertension [41]. In a therapeutic trial or epidemiological study, the probability of a true odds ratio (OR) of 2 manifesting as a significant OR of 2 in a sample may differ greatly from the probability of an OR of 2 in the population, given that the OR is significant and equal to 2 in this sample. In Dequin’s study [37], although the test is significant, it does not allow us to conclude whether hydrocortisone is truly effective or if the result is a false positive. This trial can only be classified as showing a difference in efficacy between hydrocortisone and placebo. However, we cannot draw any general conclusions about the actual efficacy of hydrocortisone compared with a placebo without additional assumptions, as we shall see later. The fact that the FDA requires two positive trials to grant marketing authorisation merely creates the illusion of classification; it amounts to adding two “+” signs to an empty table [42].

### 2.2. Fisher’s Significance Test

Fisher had a markedly different view on testing. He is known for being a biologist, agronomist, and statistician. He worked at the Rothamsted Experimental Station, an agricultural research institute, for many years. The institute’s objectives included evaluating the effect of fertilisers on crops. Fisher’s questions were mainly scientific in nature and had little to do with industrial problems. In general terms, he needed to know whether fertiliser A produced better yields than fertiliser B, regardless of whether the batch used in the experiment was classified as H0 or H1, as it would not be this particular batch that would be marketed, even if the fertiliser proved effective. Fisher required general scientific knowledge about the yield attributable to the fertiliser being studied, regardless of the type of soil on which it was used—not just the soils at the Rothamsted station. This significantly influenced the way Fisher designed his *significance test* and how he interpreted its results.

The ST proceeds as follows. After formulating a single null hypothesis H0 specifying the absence of difference between two groups to be compared, Fisher sets a risk level in the conclusion of the test. He then calculates a test statistic but converts it into a probability, the famous *p*-value. The *p*-value is the probability, if H0 is true, of obtaining data at least as far from H0 as the observed data [17,40]. This sentence is particularly difficult to understand. Nevertheless, we can attempt to clarify it. When comparing two treatments, if we observe an effect of 10 units for one and an effect of 8 units for the other, the difference is 2 units. Assuming that there is no difference in the average effect between the two treatments, the *p*-value is the probability of obtaining a difference of 2 points or 3 points or 4 points or more, and so on, in a sample. This *p*-value is then compared with a probability that has been set in advance, for example, 0.05. If *p* < 0.05, H0 is rejected, with the following argument given by Fisher: “either the observed difference is an exceptional event [because it is rare, occurring with a probability of less than 0.05], or H0 of equality of means is false” [13,17,40]. Fisher considers that the smaller the value of *p*, the higher the level of evidence against H0. Therefore, *p* = 0.01 would be stronger evidence against H0 than *p* = 0.04. However, this can only be true if these two *p*-values are calculated on the same type of data with the same sample sizes in two identical replications of the same experiment. In all other cases, two *p*-values cannot be compared, and statements such as “this result is more significant than that one” are meaningless. Why? Because one of the major flaws of the *p*-value is that it depends both on the observed difference, specific to the sample used, and on the sample size [13]. For instance, an observed difference of 2 points in a sample of 300 subjects may have the same *p*-value as an observed difference of 5 points in a sample of 10 subjects. Two studies with completely different sample sizes and effect sizes can therefore result in the same *p*-value. This demonstrates that the *p*-value is not useful for judging the difference between two groups (for judging the effect size) since it does not provide general information on the probability of achieving a clinically relevant effect. The *p*-value is like a shock absorber whose height depends both on its intrinsic resistance and on the weight applied to compress it. This also explains why, with ST, H0 is only ever a temporary conclusion, since a larger sample size could reject H0 for the same effect size. The more weight we put on the shock absorber, the shorter it becomes. Each time the tool is used, the reference frame changes.

This last point resolves the endless debates in the literature on whether H0 can be accepted definitively or only temporarily [17,18,19,20,21]: it depends on the tool used. With NHT, H0 is formally accepted, as a decision must be made on a batch. With Fisher ST, however, H0 may not be rejected, but it is only temporarily accepted (since more pressure can be applied to the shock absorber, and so on). Since the NHT is an abusive mixture of Fisher ST and Neyman–Pearson HT, there can be no clear stance on the decision regarding H0. Finally, if the acceptance of H0 is always temporary with ST, then by recursive reasoning, the test is completely useless. We know in advance that it can be made significant if we have the financial means to build a sufficiently large sample to reject a given deviation, however small.

In addition to depending on both the effect size and the sample size, the *p*-value does not depend on the true effect size in the population. If the true effect difference between treatment A and treatment B is 3 units and the observed difference in the sample is 2 units, the *p*-value is the probability of obtaining a difference of at least 2 units if the difference in the population is zero. It would be more interesting to condition the test on a value of 3 units.

Another major drawback of Fisher’s method is that it is based on an unrealistic hypothesis, namely, the null hypothesis. In its standard form, this hypothesis states that there is no difference whatsoever between the means of the two groups being compared. In a therapeutic trial, this means that the mean effect of the experimental treatment is identical to that of the control group. In other words, it is equal to zero to the 15th or 20th decimal place, which is meaningless. When different molecules are introduced into organisms, there is no chance that they will have exactly the same effect. The relevant biological question is whether the difference between the experimental and control treatments is small enough to be negligible, and whether the effects of the two treatments can be considered similar from a practical point of view.

Fisher’s significance test does not define the power of the test, nor does it formulate an alternative hypothesis [15,17]. Consequently, it is not necessary to calculate the sample size since there is no decision to be made regarding the sample, and it is therefore unnecessary to comply with classification error rates. Epidemiologists understand this well, as they work only with observational data for which H0 can ultimately always be rejected in the end, without first calculating the sample size. The issue here is not whether or not to comply with a standard, but rather to quantify the magnitude of an effect. However, in epidemiology, highlighting an OR of 1.02 obviously does not have the same meaning as highlighting an OR of 2.4, even if the associated *p*-values may be the same. Incidentally, this suggests the need to define in advance the relevant effect to be highlighted in order to test it explicitly, which is almost never done.

It should be noted that, unlike Fisher ST and NHT, the Neyman and Pearson procedure does not use a *p*-value but two predefined ranges of values compatible with either H0 or H1. It should also be noted that Fisher does not formally compare *p* and alpha [6,15,43]. Here too, appearances are deceiving. Fisher compares the *p*-value to a numerical value, which is most often set at 0.05 [43]. However, it is important to understand that this is not the alpha value specified in a Neyman–Pearson HT, but simply a preset number. It is an irony of history that led to confusion between the alpha risk of the Neyman–Pearson HT, often set at 5%, and Fisher’s 0.05 significance level. However, these two values do not represent the same thing. For the Neyman–Pearson HT, alpha is an error rate on an infinite number of tests that exhibit frequentist properties. Within Fisher’s paradigm, the 0.05 is regarded as an arbitrary numerical threshold against which the *p*-value is compared but it is not, in itself, a probability. Alpha and 0.05 are therefore not the same entities and comparing them is not relevant.

Despite appearances, the significance test of Fisher and the hypotheses test of Neyman and Pearson are very different in their approach to nature and the use of statistics (see Table 2). These two methods do not apply to the same situations, and neither is valid for therapeutic trials, epidemiological studies, or fundamental biology. Fisher’s ST is closer to the needs of scientists, but its characteristics make it ill-suited to their needs.

## 3. Beyond Significance Test and Hypothesis Test: Towards Bayesian Methods

### 3.1. Statistical Tests and Diagnostic Tests

There are some similarities between the procedure of Neyman and Pearson and diagnostic tests.

The use of a diagnostic test (DT) is based on a few simple principles. Healthy and diseased subjects are drawn from the same population in which the prevalence of the disease is supposed to be known. To determine whether a subject has a certain disease, a test with known sensitivity and specificity is performed. If the test performed on the patient is positive, the probability that the subject has the suspected disease (the positive predictive value, PPV) is calculated using Bayes’ theorem. It is important to note the difference between having a positive test result Pr(T+) and actually having the disease when the test is positive Pr(M+|T+), i.e., the PPV, as these are often confused.

A parallel can be drawn between the diagnostic test and the Neyman–Pearson HT. By equating a compliant batch (H0) with being healthy and a non-compliant batch with being diseased, sensitivity is equivalent to the power of the test, and lack of specificity is equivalent to alpha risk (Table 3). Another similarity should be noted: in both tests, the intrinsic performances are known or considered as such. This means that the sensitivity, specificity, alpha, and beta are the respective properties of these tests, and their values are known or defined before using either test. However, this parallel between the two tests is only valid to the extent that the DT allows the PPV of a positive test to be calculated given the prevalence of the disease. In the Neyman–Pearson HT, if the test is significant, it is not possible to calculate the probability that H1 is the correct hypothesis (i.e., a PPV) because the a priori probability H1, which would be equivalent to the prevalence of the disease, is missing. This results from Neyman and Pearson’s choice not to use Bayes’ theorem, but this prevents them from calculating the probability that a batch classified as H1 (non-compliant) is actually of type H1. As outlined above, this is the root cause of the paradox of the Neyman–Pearson HT. The diagnostic test requires at least an estimate of the prevalence of the disease to calculate the PPV. Similarly, to calculate the probability of a batch being non-compliant, given that the test is significant, the table must be completed and an additional row added to the last row of the HT table: the a priori probabilities of a batch being compliant or non-compliant must be specified in order to calculate the “PPV of non-compliance” when a batch test is positive and classified as non-compliant. Table 3 shows the addition of these probabilities in the lower margin of the table. However, it is important to interpret these probabilities correctly. In this example, the lower margin (greyed out) indicates that 700 and 300 batches are compliant and non-compliant, respectively, giving an initial probability of 0.7 and 0.3 for H0 and H1. How should these values be interpreted? They cannot be interpreted as the frequencies of the true null and alternative hypotheses, because then, as with the diagnostic test, this would assume that we already know the “prevalence” of H0 and H1, i.e., the probability that each hypothesis is true. It would therefore be illogical to attempt to establish what we already know. The primary responsibility of the researcher is to establish this knowledge and to determine the validity of each hypothesis. In other words, the aim is to determine the probability that treatment A is more effective than treatment B for the target population.

How can we then properly interpret this “prevalence”, or rather, this probability that H1 (or H0) is true? To address this issue, it is first necessary to properly define the concept of probability.

### 3.2. The Three Interpretations of Probability

The classic definition of probability states that it is the ratio of the number of cases in which the event of interest occurs to the number of possible cases. This is the definition used in the very classic problems of dice rolling and card games. This definition has the advantage of being usable for single events and calculable a priori (I roll a single die once; I can calculate the probability of obtaining a 3 before rolling the die). The value of this probability is logically accessible: the probability of a given event can be computed without actually throwing the (fair) dice. However, this approach is circular, as it assumes that events are equiprobable, which requires the probability to have been defined beforehand. If we use a non-homogeneous die, made of wood or bone, how could we calculate this probability when the argument of equiprobability of the sides of the die clearly does not hold? This is a functional definition that is applicable in certain situations, but it is not general and does not in itself define what probability is.

Another definition, called frequentist, states that probability is the asymptotic value of the ratio of the number of times the event of interest has been observed among all the situations in which it could have occurred. This is how we calculate the probability of contracting influenza or experiencing an adverse event during treatment. The value of this probability can be obtained empirically. However, it should be noted that the disadvantage of this definition is that the probability obtained in this way is always known a posteriori, as it can only be calculated on events that have already occurred. This definition is also circular: for the definition to be valid, events must be equiprobable, which is rarely the case in medicine. Finally, it cannot be calculated for a single, yet uncertain, event: what is the probability that Mrs Z will experience an adverse effect when taking treatment T, or that it will rain in Tokyo on 27 September 2070? It is not possible to obtain a frequentist a priori estimate of these probabilities. Ultimately, this definition does not provide a mean to calculate the probability of a theory being true. For instance, it is not possible to determine the probability that treatment A is more effective than treatment B for patients being treated for community-acquired pneumonia—and no, the *p*-value is not the answer to this question.

This digression allows us to introduce a third definition of probability: the subjectivist definition. It is common to say of a confidence interval that there is a 95% probability that the value of the risk difference will be, for example, between 1.7 and 9.6. Contrary to appearances, this very common formulation used in almost all current publications is not valid [3,44,45]. This is one of the many counterintuitive characteristics of these methods. The classic confidence interval is not an interval with a 95% chance of containing the true value of the parameter of interest in the population. To understand why, consider the following example: if we were to conduct exactly the same trial twice at the same time, under the same conditions, with different subjects but all from the same population, for example, to estimate the difference in treatment effect, the sampling would give two different estimates of this difference, with two different confidence intervals. It is not possible for these to simultaneously have a 95% chance of containing the true value of the delta difference. Another example may be useful: suppose that a serious adverse event (SAE) is observed among 37 patients out of a sample of 100 subjects. The estimated SAE rate is therefore 37% [27.7–47.3]. The prevailing interpretation is that there is a 95% chance that the true SAE rate is between 27.7 and 47.3%. However, if we had observed 200 other subjects and assumed that we had 74/200 SAEs, we would also have an SAE rate of 37% but with a confidence interval of [30.4–44.1], which we would also say has a 95% chance of containing the true value of the SAE rate. However, it is mathematically impossible for both of these intervals to contain the true value of the SAE rate (that of the population) with the same probability since they are not the same length. The first interval is significantly wider, “spilling over” more than 5% of the second interval and resulting in a total width of more than 100%. This difficulty is not obvious, as it is uncommon to calculate the same confidence interval twice on different sample sizes within the same study, but meta-analyses provide a compelling illustration of this phenomenon. Each trial has its own confidence interval for the difference between A and B. Consequently, it is impossible for all these intervals to concurrently possess a 95% probability of containing the true value of the difference. This example illustrates that the conventional interpretation of the confidence interval is not correct. The correct frequentist interpretation is as complicated to understand as the correct definition of the *p*-value. A 95% confidence interval is an interval such that if an infinite number of intervals are constructed in the same way in the same population, 95% of them will contain the true value of the parameter under study [46]. This complex, counterintuitive yet rigorous definition has the advantage of being consistent with the notion of iterative sample classification, as performed by Neyman–Pearson HT.

To interpret a confidence interval in its usual context, the interpretation of the concept of probability must be modified. Using the example of the SAE rate, since the second sample is larger than the first, it is logical to say that in the second interval, 95% of our knowledge of the SAE rate is contained in the interval [30.4–44.1], whereas in the first sample, our knowledge was vaguer since it was based on a smaller number of subjects. The true value of the SAE rate in the population has remained constant, but our knowledge of this value has increased because the second SAE rate estimate is based on a larger sample, or rather on additional knowledge. This probabilistic knowledge is referred to as subjective or subjectivist probability, or epistemic probability, which relates to the judgement we make about the value of a parameter. With this third definition of probability, probability is interpreted as the level of credibility that can be attributed to a series of values for a parameter or as the amount of knowledge available about the parameter. This subjectivist interpretation of probability is the only correct interpretation that can be used in a probabilistic expression of an effect size (a difference in proportions or a correlation coefficient) to be estimated in a population. It is important to note that the knowledge of the value of an OR or a mean difference in the population is necessarily subjective since it is not directly accessible. It can only be calculated on observed data. The value of probability in this situation is therefore only accessible through understanding and knowledge, even though this knowledge may be guided by empirical data. The subjective definition of probability is the only one that allows us to assess the probability that, in the population to be treated, treatment A is superior to treatment B. This is a judgement on the veracity of the fact that A > B. However, this probability should not be confused with the *p*-value. The *p*-value is the probability that in a given therapeutic trial, the observed proportion of subjects responding to A will be greater than the observed proportion of subjects responding to B, under the sole assumption that A and B have exactly the same effect.

Let us return to the example of the SAE rate. The subjectivist interpretation of probability has the advantage of ensuring consistent interpretation of the two SAE rate intervals, despite their different values. This interpretation suggests that each interval quantifies the knowledge available *at the time it is stated, based on the data available*. Therefore, we can conclude that each interval is a 95% credibility interval, i.e., an interval of values that contains 95% of the available knowledge about the parameter of interest at the time it is calculated, with our knowledge limited to the information available at that time about this sample. Once we have knowledge of both samples, it becomes possible to merge the knowledge obtained from each of them to obtain a third, even narrower interval that will describe 95% of the information available from 300 subjects. This subjectivist interval therefore also necessarily refers to the value in the population of interest and not to the value observed in the sample, which nevertheless contributes to the construction of this interval by providing empirical knowledge that modulates subjective knowledge. It should be noted that the erroneous interpretation of the frequentist interval is quite similar to the Bayesian interpretation of the confidence interval. This is reassuring for clinicians, whose intuition is often sound, even if it does not conform to classical statistical theory: the more one learns, the more one knows.

It should be noted that the aforementioned three definitions of probability are in no way contradictory. All three are mathematically valid in their own field of application, as they all satisfy the axioms of probability established by Kolmogorov. Only the situations in which they are applied differ (see Table 2).

Return to Table 3, we established that the margin of the table (700; 300) cannot be interpreted as frequencies, which would assume that these frequencies are already known. It must be interpreted as the a priori credibility assigned to hypotheses H0 and H1, with Bayes’ theorem applied via the subjective probability that each hypothesis is true. The margin in the table quantifies the level of credibility assigned to each hypothesis prior to data collection from the experiment conducted to test (this time for real) the two hypotheses.

### 3.3. Performing a Test with Bayes’ Theorem

There is a need to refine this approach, with the ultimate objective being to determine how this concept of subjective probability can be utilised to perform a statistical test by applying Bayes’ theorem [26,27,28,32].

**Figure 1:**
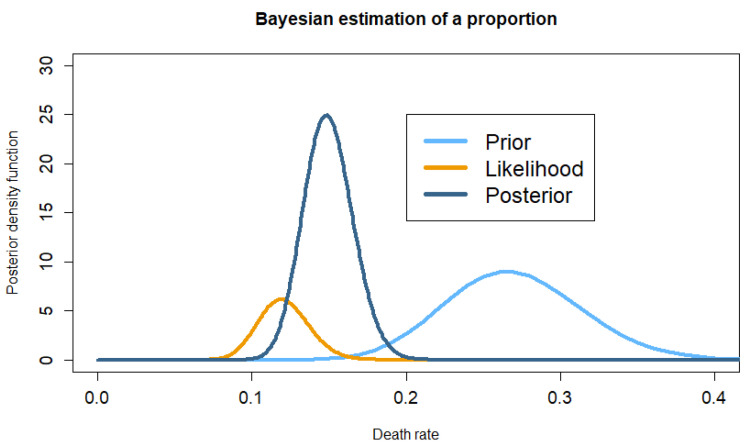
Illustration of the Bayesian estimation of a proportion. The prior distribution is the mathematical expression of the knowledge available on the proportion to estimate before the trial. The prior used here expresses a knowledge equivalent to 27 deaths out of 100 subjects. The likelihood is the probability of the observed data for each possible value of the proportion. The posterior distribution is the mathematical translation of all the knowledge available about the proportion after the trial is run.

When calculating the sample size, Dequin estimated the death rate in the placebo group to be 27%. This initial value of 27% is obviously not known with perfect accuracy and must be accompanied by a subjective confidence interval. However, it is nevertheless clinical knowledge based on the experience of the researchers who developed the study, and it is the numerical representation of established clinical knowledge. Let us assume that this rate has an initial (prior) subjective probability distribution of 95% of falling within the interval [18.8–36.1]. The observed death rate in the trial was 47/395 (11.9% [9.0–15.6]), which adds to the prior knowledge of 27%. Figure 1 illustrates this prior knowledge, as well as the knowledge provided by the data. These two sources of knowledge are mixed via Bayes’ theorem to form the final knowledge on the death rate, summarised in the posterior probability distribution. The Bayesian estimate of the death rate in the placebo group at the end of the trial was 14.9% [12.0–18.2], which differs from the empirical estimate based on the sample. This is not surprising, as the death rate observed in the placebo group of the sample has virtually no chance of being exactly equal to the rate in the population, and this rate is best known by the final Bayesian estimate, which combines prior knowledge and objective data.

The same principle applies when comparing the two treatment groups. To proceed, it is necessary to express the prior knowledge of the death rate in each group used when calculating the number of subjects required. This will allow prior knowledge on the difference in death rates to be expressed. The difference in death rates between the two groups will be analysed using both prior knowledge and the empirical knowledge provided by the observed difference in the sample (25/400–47/395). At the end of the study, subjective posterior knowledge about the difference in death rates in the population of interest will have been established. This difference of −5.9% [−10.0–−1.9] favours corticosteroid therapy. The mathematical details of this calculation are beyond the scope of this article but are relatively simple.

These two examples illustrate all the elements necessary to perform a Bayesian analysis of a therapeutic trial, i.e., the prior distribution, the likelihood, and the posterior distribution.

The prior distribution of the parameter of interest (difference in proportions, means, etc.) is the set of credible values of the parameter under study and their respective probabilities.

The probability of the observed value of the parameter in the sample is dependent on each possible value of the estimated effect. In Bayesian terminology, this is known as the likelihood, i.e., the probability of the data for each possible value of the parameter defined in the prior distribution.

The posterior distribution is derived from calculations using the Bayes theorem and represents the set of credible values for the parameter under study and their respective probabilities, based on the data. It can be used as a prior distribution for a subsequent update of the parameter distribution.

The combination of these three elements is crucial for the acquisition of knowledge. The example of the death rate in the placebo group demonstrates the application of the Bayesian process. Starting from limited knowledge about a parameter, the empirical acquisition of data in a trial will increase knowledge about that parameter. Bayes’ theorem is simply a mathematical tool for acquiring knowledge in situations where there is uncertainty. Initial subjective a priori knowledge will be transformed into subjective a posteriori knowledge thanks to the empirical knowledge provided by the experimental data. The process is straightforward: it involves updating and reallocating probabilities across all possible values of the parameter (e.g., odds ratio, difference in risk or means, etc.). Bayes’ theorem can be written as follows, for example, for a delta difference and data D of a sample:

Pr(delta|D)∝ Pr(delta)·Pr(D|delta)

In everyday language, this can be expressed as follows: the knowledge about delta in the population after observing the data (Pr(delta|D)) is proportional to the knowledge about delta before the experiment (Pr(delta)) and to the knowledge provided by the sample data (Pr(D|delta)). Even more simply: what I know after the trial depends both on what I knew about the difference between A and B before the trial and on the data observed during the trial.

It is important to acknowledge that probability is a measure of uncertainty regarding the value of a parameter and, as such, can be considered a quantification of knowledge. This understanding enables us to combine the information from two successive clinical trials [47] and accumulate the knowledge gained from both trials. The Neyman and Pearson procedure and the Fisher method do not allow for this, as they only consider the probability of the data observed in the sample. They do not consider the probability of the phenomenon in the population that generated these data. For philosophical reasons, Neyman and Pearson did not want to use the subjective probabilities of Bayesian methods. They designed a test that did not require specifying any prior probability of the two hypotheses being compared, relying solely on the frequentist probabilities of the observed data in iterative tests. Their procedure is therefore unable to estimate the probability of these two hypotheses from the observed data. Consequently, it cannot accumulate knowledge about these hypotheses even considering several similar clinical trials. This is counterintuitive, as each researcher ends up convincing themselves that across trials, treatment A is more effective than treatment B by accumulating observations. Researchers are often naturally Bayesian, starting with limited knowledge of the effects of treatments and updating this knowledge using the results of different trials, thereby reducing their uncertainty. However, by using *p*-values, they prevent themselves from making scientific observations and manipulate the frequentist tool to make it say what they want. “What’s wrong with NHT? Well, among many other things, it does not tell us what we want to know, and we so much want to know what we want to know that, out of desperation, we nevertheless believe that it does!” [7].

### 3.4. The Interpretation of Trials in Terms of Knowledge Accumulation

In the case of hydrocortisone use to treat community-acquired pneumonia, the observed risk difference was –5.6% [–9.6 to –1.7]. A Neyman–Pearson HT would classify this result as being compatible with hypothesis H1, but it would not be possible to draw any general conclusions about the effect of hydrocortisone, as discussed earlier. A Fisher ST would add a *p*-value of 0.006 and reject the null hypothesis of no difference. However, this *p*-value would not indicate the actual importance of the effect on the population to be treated. What would the Bayesian interpretation be? With reasonably informative prior distributions, i.e., with a reasonable amount of previous knowledge, we obtained a difference in death rates of –5.9% [–10.0 to –1.9], indicating that based on our current knowledge, the difference in risk is negative and lies between –10% and –1.9%. This suggests that the benefit is not zero (as 0 is excluded from the credibility interval—the Bayesian equivalent of the confidence interval). However, care must be taken not to interpret this result in the same way as a Neyman–Pearson HT, which only considers sample data. This is an estimate of the effect in the population. It is not the observed or observable frequency of the difference across multiple samples, but rather the degree of knowledge attributed to the studied difference. In other words, once the trial is run, 95% of my knowledge indicates that the difference lies between −10 and −1.9%. Finally, it should be noted that a classical confidence interval can be numerically identical to the Bayesian credibility interval when the latter is based on uniform prior distribution in which the prior knowledge specifies that all values of the parameter have the same probability. Nevertheless, the interpretations are still different.

For a full picture, the probability of a clinical benefit should also be calculated. Based on the values used to calculate the sample size, the death rates should have been significantly higher at around 20.25% and 27%, i.e., a relative risk of 0.75. The observed rates and relative risk (0.42) were substantially lower. Interestingly, Dequin calculates the sample size for a relative risk, yet presents the outcome as a risk difference. The relative risk (RR) estimated by a Bayesian method is 0.60 [0.42–0.85], which is higher than the observed RR. Decisions must ultimately be made, particularly regarding the future administration of cortisone to patients with community-acquired pneumonia. To inform this decision, it is sufficient to calculate the probability that the difference will favour hydrocortisone. For example, we can calculate the probability that the 25% relative reduction used to calculate the sample size will be achieved in the population to be treated. Using simple Bayesian methods, we show here that there is an 89% chance that the RR is actually lower than the expected value of 0.75.

Another investigator may want to bring less prior knowledge to the analysis to obtain an estimate based primarily on the data. In this case, they would use a uniform prior. The difference in risk would then be −5.6 [−9.7, −1.7], the relative risk would be 0.53 [0.33, 0.83], and the probability of an RR < 0.75 would be 0.93.

The use of different prior distributions yields different posterior results. In Bayesian statistics, it is common practice to perform sensitivity analyses using different prior distributions—not to find the one that produces the desired result, but to estimate the extent to which the information provided by the data is quantitatively significant in relation to prior knowledge.

If the results differ greatly for small variations in the prior distributions, this essentially means that more data needs to be collected. This is not an estimation error or data dredging, but rather an explicit consideration of the fact that the prior distribution is an integral part of the model and that the model is an integral part of the estimation process.

## 4. Benefits of a Bayesian Interpretation of Trials

### 4.1. Sequential Trials

In a sequential trial, the statistical analysis of the primary endpoint is performed several times rather than once on all the data collected. These analyses take place at intermediate stages, the timing of which must be specified in advance according to Neyman and Pearson. As the same analysis is performed several times on partially correlated data, the alpha error risk must be adjusted so that it remains at its nominal value (usually 5%) across all tests. The greater the number of tests performed on a given sample, the greater the risk of obtaining at least one significant result. This risk is therefore to be corrected, and there are several methods of doing so. However, these corrections do not prevent paradoxical conclusions from being reached [48]. For instance, it is possible for a final analysis that would be significant with *p* = 0.04 to become non-significant when an intermediate analysis is added because of a decision threshold of 0.0253 to maintain the overall alpha at 5% [49]. In the case of sequential testing with non-Bayesian methods, the risk of error is changed in classifying this sample at the time of each interim analysis, but no additional knowledge about the population is gained. When using a Bayesian method and interpreting probability as the amount of knowledge about the parameter available at the time of analysis, the final analysis pertaining to the population always yields the same conclusion, regardless of whether one, ten, or a hundred intermediate analyses are performed. This is because the final knowledge is always the same.

### 4.2. Meta-Analyses and the Accumulation of Knowledge

Meta-analyses (MAs) have become very common. Beyond their inherent limitations, which can result in two MAs being completely contradictory [50], there is an apparent paradox in MAs that may be surprising. Consider an MA based on 30 very similar therapeutic trials. One might wonder why the 30th trial was undertaken when the first studies had already been published. This creates the curious impression that the proliferation of therapeutic trials does not provide any additional information compared with previous ones. Forest plots of MAs perfectly illustrate the spirit of Neyman and Pearson’s tests: each trial is individually classified as either “significant” or “not significant”, without actually testing this difference at the population level. No real learning takes place about the value of this difference. Consequently, the accumulation of data does not lead to an accumulation of knowledge, preventing us from drawing an overall conclusion about the difference in effect between A and B. Lau [47] described this paradox well, referring to the concept of a cumulative meta-analysis (MA), which involves adding each new study to the MA by updating it. This results in an increasingly narrow confidence interval and an increasingly accurate estimate of the difference in effect between the two treatments. These cumulative MAs significantly reduce the number of trials and patients needed to reach a usable conclusion and resolve the aforementioned paradox if probability is interpreted as a measure of knowledge about an estimated effect: the more knowledge accumulated about the effect, the more accurate the estimate becomes. However, there is only one estimate, which changes as knowledge accumulates.

### 4.3. Multiple Comparisons

Should the alpha risk be adjusted to account for multiple comparisons? This is another great mystery of statistics. If we test 20 variables for which there is no real difference between the two groups being compared, there is a 64% chance that at least one of the tests will wrongly show significant results. This largely exceeds the accepted alpha risk of 5%. As with sequential trials, there are various methods of alpha risk correction in the literature. The best known of these is undoubtedly the Bonferroni method, but there are several others. However, these corrections also regularly lead to paradoxical or contradictory conclusions. One major problem is that the correction depends on the number of variables tested, meaning the result for one variable depends on whether another variable has been tested. A variable may therefore be significant when five variables are tested, but not significant when 20 variables are tested [48]. This makes no sense unless we remember that these corrections were developed for Neyman and Pearson’s HT, which is used to classify batches according to a couple of hypotheses. The aim is to avoid classifying a batch as defective when it is not, since a batch can be evaluated on several characteristics simultaneously, each of which must meet a quality standard. A batch will be destroyed as soon as quality control reveals a defect in at least one characteristic (under the so-called universal null hypothesis [48]). In a clinical trial, if we accept that each test provides knowledge about a variable, it is difficult to see how knowledge about a given variable could depend on whether another variable is tested. For example, this suggests that the average birth weight of a child with a genetic mutation could be considered different from the average weight of normal children if only birth weights are compared, but not if calcium and bilirubin levels are also considered, even though the average weight would obviously be identical in both cases. This makes no scientific sense. Again, interpreting probability as a measure of knowledge about the parameter allows paradoxes and ambiguities to disappear.

### 4.4. Hierarchical Tests

Hierarchical tests [51,52,53,54] are a method of managing alpha risk when, as in most therapeutic trials, treatments are compared on several endpoints, which increases the probability of having at least one significant test, even under the assumption that there is no real difference between the two treatments being compared. The method involves establishing a hierarchical list of endpoints to be compared, starting with the primary endpoint and then ranking the secondary endpoints in descending order of relevance. The primary endpoint is tested, and if it is significant, the first secondary endpoint is then tested. This process is continued as long as the tests prove significant. As soon as a test is non-significant, the criteria lower down in the hierarchy are not tested. This ultimately prevents the acquisition of knowledge, even from a Neyman–Pearson perspective, on all the criteria lower down in the hierarchy, despite the energy invested in collecting these criteria. Aside from the financial waste that this procedure represents, the scientific value of this method is difficult to discern. If an alternative order had been used in the hierarchy, an untested criterion could have proved significant, and conversely, a significant criterion could be considered insignificant in another hierarchy simply because it was placed after a criterion that proved insignificant. This approach appears to lack coherence. By interpreting probability as the amount of knowledge available about the value of a parameter, it appears that each secondary outcome can and must be estimated on its own merits, and that the desire to acquire knowledge about a certain criterion should not depend on the estimated effect on another outcome. For instance, it would be difficult to comprehend from a scientific perspective the refusal to evaluate the performance of chemotherapy in terms of recurrence-free survival on the basis that it is not significantly different from a reference treatment in terms of overall survival, when this assessment is made using a tool, i.e., the *p*-value, that is incapable of judging this. The issue is not whether to classify a therapeutic trial according to a list of criteria, which is likely to be truncated during the process, but to acquire knowledge about each of these criteria on the studied treatment in the interests of patients. If the knowledge is not precise enough, for example, to ascertain whether the risk of recurrence is genuinely reduced, we will at least be aware of the order of magnitude of the effect and have a rough idea of the number of participants to be recruited to achieve the desired precision.

## 5. Discussion

Biology and medicine are essentially observational sciences, and thus, it is very rare to be able to establish a predictive and testable model, as can be achieved in physics. To establish the laws of biology and acquire knowledge that is, by nature, necessarily partially random, it is necessary to resort to observation and sampling, which also involves the use of probabilities. However, this can only be achieved with an interpretation of probability that is adapted to the subject of this science. Frequentist probability is applicable when events can have a physical existence, such as a sample of patients for which the observed OR can be calculated. Subjective probability is used when the uncertain event to be evaluated cannot have a physical existence, cannot be materialised, is inaccessible, or is unique, such as the OR value for an entire population. In a clinical trial, a sample is obtained, and the specific probabilities of each statistical test, whether it is an NHT or a Fisher ST, relate to the data obtained (Fisher ST) or the procedure used (Neyman–Pearson HT) but not to the hypotheses. The purpose of science is to establish the veracity of a theory and therefore of the plausibility of the underlying hypotheses; the probability of the data is only a way to evaluate this plausibility. When considered in isolation, trial data provide no information on the plausibility of hypotheses. The goal is to obtain a neutral and objective result on the value of interest in the population—a result that would be guaranteed by the observation of the figures alone, without the subjectivity of the researcher. However, the random and variable nature of samples prevents us from ever achieving a perfectly objective result for the population. When the value of an unknown parameter cannot be deduced from a theory or theorem, obtaining its value ultimately relies on subjective probabilities. As James Maxwell put it, “[...] the true logic for this world is the calculus of probabilities, which is, or ought to be, in a reasonable man’s mind” [55]. We are obliged to make a judgement on the credibility of the parameter values, which is necessarily subjective (but not arbitrary) and involves probability. However, the probability assigned to each value cannot express frequency since the parameter in the population is unique; it is neither random nor probabilistic in itself. The subjective probability distribution associated with this parameter, i.e., the assumptions about the plausible value of this parameter, which is neither repeatable nor observable [56], serves as a measure of our knowledge of it.

While subjective probability is perhaps more of a conceptual framework than a practical reality, it is nonetheless a valuable concept, much like triangles, points, or complex numbers. It is important to note that a mathematical triangle, i.e., a perfect triangle, has never been observed. Nevertheless, it is a concept that is particularly fruitful in a wide range of everyday applications.

It is equally important to consider the psychological and cognitive aspects when assessing the long-term popularity of a tool that is not well-suited for research, such as the NHT. It is evident that a variety of biases, including confirmation bias and selection bias, are likely contributing to the ongoing misapplication of statistics [57].

Despite their almost systematic use, statistical tests and *p*-values are misused, misunderstood, and poorly taught [58,59,60]. As outlined above, the vicious cycle may be broken by improving the teaching of statistical methods. It is noteworthy that few courses address the two forms of the non-Bayesian test. This is probably at least partially why so few, including clinicians [61] and even sometimes statisticians, understand or use them correctly [62]. It is a common misconception that there is only one tool, when in fact there are two different tools, each with its own specific function [63]. This also prevents teaching that the test that everyone currently uses is a procedure that does not exist and which, by mixing what cannot be mixed, results in a completely faulty procedure. However, the persuasive power of the substantial user base of this test is such that each user must either relinquish any attempt to comprehend these methods in depth or, perhaps even more problematically, deceive themselves into believing they understand how it works.

Conventional tests are still very widely used in the current literature, and many authors have presented arguments in favour of maintaining these tests as a basic statistical tool for analysing data from therapeutic trials or epidemiological studies. Some even go so far as to claim that abandoning conventional tests would be detrimental to scientific practice [64]. Most, however, recognise the limitations of conventional tests and that the level of evidence provided by the NHT is insufficient. Many propose solutions to circumvent these limitations. Without being able to cite them all, it can be seen that most approaches revolve around the very specific difficulties of interpreting conventional tests and emphasise the use of complementary indices, such as effect size measures or confidence intervals [65].

Benjamin proposed enriching the interpretation of *p*-values, which he believes are not about to disappear from the literature, through various data analysis practices, including interpreting *p*-values in the context of tools derived from Bayesian statistics, such as the Bayes factor [66].

Some authors point out that while classical tests do indeed have limitations, several of the proposed alternatives, such as the use of confidence intervals instead of *p*-values or Bayes factors, do not offer real solutions to the criticisms levelled at classical tests, indirectly justifying their use in their standard form [67].

Others, without proposing alternatives, call for a more nuanced and reasoned use of conventional tests, based on a better understanding of what these tests can actually do [68]. Greenland emphasises that *p*-values behave as they should, but the way users apply these tests may need review, suggesting supplementing user training with an improvement in their scientific thinking through information and surprise indices [69]. Along the same lines, several authors suggest that the correct use of tests, through improved training of researchers in scientific methods, would improve the interpretation of data processed by frequentist tests (Laken) [64,70,71]. Meehl also believes that the problem is not so much the intrinsic limitations of *p*-values and tests as their misuse by scientists who are insufficiently trained in scientific method and epistemology. To this end, he distinguishes between the concepts of substantive theory and statistical hypothesis [72].

It should be emphasised that frequentist methods are mathematically valid when interpreted correctly. It is their characteristics, which are very subtle and little known to their users, that largely explain the many errors of interpretation to which they are subject. They are also limited by their poor ability to assess the veracity of a hypothesis. However, we cannot blame them for this, as they were not designed for this purpose. To this end, while remaining within the classical framework, several authors have proposed alternatives to the Neyman–Pearson and Fisher methods. We can cite Fisher himself, who proposed another form of test, known as fiducial inference [73]. More recently, others have proposed and developed severe testing and error statistics as a method of circumventing the limitations of classical methods [74,75,76]. Although these solutions have been widely discussed in statistical and philosophical literature, it must be noted that they have not been very successful and remain virtually unused.

Most readers will probably find it challenging to accept the message conveyed here. This represents a substantial shift in the way statistics are perceived, and the cognitive dissonance that the average user experiences after utilising NHT for so long and in considerable amounts may prove to be significant. It may be challenging for them to come to terms with the extent to which they have been misled. It may take time for readers to assimilate the information in this article, but if they think about it every time they encounter a *p*-value in the future, they will be able to break out of the vicious cycle. Once the Bayesian principle is understood, the book of science becomes much more readable, and it is difficult to go back to previous texts.

## 6. Summary

The Neyman and Pearson HT classifies a given sample into one of two categories, according to two hypotheses, with pre-defined error rates. These error rates remain constant regardless of the prior probabilities of the two hypotheses. Therefore, the Neyman–Pearson HT is not informative about the probability of each hypothesis.

Fisher’s significance test quantifies the difference between the observed and expected data under the null hypothesis of no difference, completely independently of the probability of this hypothesis. Therefore, the Fisher ST is not informative about the probability of this hypothesis. Furthermore, in most cases, this null hypothesis has no a priori chance of being true. The only relevant question is whether the data favour a noticeable difference between the groups, but the *p*-value does not answer this question.

Both tests are based solely on the probability of the data.

The Bayesian test is based on a subjective definition of probability that expresses knowledge about a value that cannot be accessed directly (e.g., the odds ratio in a population of subjects to be treated). It provides an estimate of the subjective probability of a hypothesis regarding the effect of a treatment in a given population based on limited prior knowledge and data observed in one or more trials.

## Figures and Tables

**Table 1:** Number of significant and non-significant tests using the Neyman and Pearson procedure in two different situations: (A) a large proportion of compliant batches; (B) only 50% of compliant batches.

	Situation A			Situation B		
	Compliant Batches	Non-Compliant Batches	Total	Compliant Batches	Non-Compliant Batches	Total
*t* > threshold, significant test	49	16	65	25	400	425
*t* < threshold, not significant test	931	4	935	475	100	575
	980	20	1000	500	500	1000

**Table 2:** Characteristics of different types of statistical tests used in biomedical literature.

	Null Hypothesis Test	Neyman and Pearson Hypothesis Test	Fisher’s Significance Test	Bayesian Test
Assumptions	H0/H1	H0/H1	H0	Not an assumption but knowledge on (a distribution of) parameter values
What the test does	Ill-defined	Classification of the sample tested	Scientific hypothesis evaluation	Scientific hypothesis/by estimating parameter value
Criteria	*p*-value	Test statistic *t*	*p*-value	Posterior distribution
Comparison criteria	α (usually 0.05)	Test statistic threshold	Numeric value (usually 0.05)	None: Description of the a posteriori distribution or threshold value if test
Decision according to	*p* < α	*t* > *t* threshold	*p* < 0.05	Credibility of a set of values on the parameter
Sample size	Unclear (often considered required)	Required	no	Possible if required
α or Type I error risk	+	+	+	-
β or Type II error risk	+	+	-	-
δ or effect size	±	+	-	-
Type of probability involved	Frequentist	Frequentist	Frequentist	Subjectivist
Object of probability	Data and parameters	Sample data and parameters	Sample data and parameters	Parameter in the population

**Table 3:** Likeness of the Neyman and Pearson procedure and the diagnostic test. Sensitivity (Se) and Specificity (Sp) are estimated and known beforehand while α and β are decided upon before the test is run. The diagnostic test classifies a subject as diseased or non-diseased, but to compute the Predictive Positive Value (the probability that a subject classified as diseased is indeed diseased) requires knowledge of the disease prevalence and the Bayes theorem. The lower margin for the quality control situation is greyed out to show where knowledge about hypothesis initial probability should be added.

	Quality Control			Diagnostic Test	
	H0: Compliant Batches	H1: Non-Compliant Batches		H0: Non-Diseased	H1: Diseased
z > threshold, significant test	α	1 − β	Test positive	1 − Sp	Se
z < threshold, not significant test	1 − α	β	Test negative	Sp	1 − Se
	700	300		1 − prev.	prev.

## Data Availability

This article does not refer to any specific database. All data are included in the text.

## Glossary

DT	Diagnostic test
FDA	Food and Drug Administration
HT	Hypotheses test
H0	Null hypothesis
H1	Alternative hypothesis
MA	Meta-analysis
NHT	Null hypothesis test
OR	Odds ratio
RR	Relative risk
SAE	Serious adverse event
ST	Significance test
PPV	Positive predictive value
